# Enzymatically Extracted Apple Pectin Possesses Antioxidant and Antitumor Activity

**DOI:** 10.3390/molecules26051434

**Published:** 2021-03-06

**Authors:** Agnieszka Wikiera, Maja Grabacka, Łukasz Byczyński, Bożena Stodolak, Magdalena Mika

**Affiliations:** Department of Biotechnology and General Technology of Foods, Faculty of Food Technology, University of Agriculture in Krakow, ul. Balicka 122, 30-149 Kraków, Poland; maja.grabacka@urk.edu.pl (M.G.); lukasz.byczynski@urk.edu.pl (Ł.B.); bozena.stodolak@urk.edu.pl (B.S.); magdalena.mika@urk.edu.pl (M.M.)

**Keywords:** pectin, enzymatic extraction, ferulic acid, antioxidant activity, anticancer activity

## Abstract

The biological activity of apple pectin extracted conventionally or enzymatically using endo-xylanase and endo-cellulase, was tested in vitro. The analyses were performerd in tetraplicates and the statistical significance of the differences were assessed using ANOVA, Tukey post hoc and LSD (the least significant difference) tests. Multivariate regression analysis was applied to determine the structural components that have a crucial importance for antioxidant and antitumor properties of pectins. The pectins extracted by enzymes contained up to four times more ferulic acid and showed twice as great ability to neutralize free radicals and Fe(III) reduction. The antiradical potential positively correlated with phenols, fucose and rhamnose content. In the assays performed on HT-29 human adenocarcinoma and B16F10 melanoma cell cultures, the “green” pectins, contrary to acid isolated ones, exhibited remarkable anti-neoplastic potential while being nontoxic to nontransformed L929 cell line. The pectins in the dose of 1 mg/mL were capable of inhibiting adhesion (max 23.1%), proliferation (max 40.4%), invasion (max 76.9%) and anchorage-independent growth (max 90%) of HT-29 cells (significance level *p* < 0.001). These pectin preparations were slightly less active towards B16F10 cells. The enzyme-isolated apple pectins may be useful as a functional food additive and an ingredient of the ointment formulas for post-surgical melanoma treatment.

## 1. Introduction

Pectins are complex plant polysaccharides and the diversity of their structures depends largely on their origin. The pectin structure is a crucial factor that determines the application in the food and pharmaceutical industry. Pectins are frequently used as gelifying agents in jams and jellies, thickeners, emulsifiers and stabilizers in dairy products and mayonnaise, dressings and margarines, or as fat substitutes in confectionery and ice creams, as well as to improve the mechanical properties of protein films and coatings [1]. Commercial pectins are usually extracted from citrus peel and apple pomace by hot acid (HCl, H_2_SO_4_ and HNO_3_) method at pH 1–3 for 1–12 h at 70–100 °C [2]. The yield of such a process is usually satisfactory, but acid extraction is energy-consuming and increases the risk of accelerated corrosion of production line elements, as well as generates large amounts of acidic industrial waste [3]. Therefore, the wider application of enzymes, which are much more selective catalysts, is encouraged to replace mineral acids [3,4]. Obviously, the choice of extraction methods has a significant impact on the composition of the obtained polymer preparation, and subsequently its functional features and further applications. For example, a pectin product isolated enzymatically is significantly different from conventional pectins in terms of molecular weight, galacturonic acid and neutral sugar content, as well as the degree of methylation [4,5]. The structural characteristics and composition of a pectin polymer greatly contribute to its biological activity. Some reports revealed that pectins, particularly highly methylated, decrease the cholesterol, triglyceride, phospholipid and free fatty acid levels in blood plasma and tissues [6]. Interestingly, pectin preparations taken with diet are able to modify the lipoprotein profile and even induce atherosclerosis regression in the already affected patients [7]. Pectins have also been shown to reduce glucose bioavailability and reduce the insulin plasma concentration, which prevents the development of insulin resistance [7]. Additionally, the pectins with low degree of methylation efficiently chelate metal ions [8]. Low methylation degree and the presence of long unbranched oligogalacturonate segments indicate the capability of a strong adhesion to the mucin layer in the gastrointestinal tract, which protects the epithelium from the invasion of opportunistic microflora [9]. The in vitro and in vivo studies also demonstrated immunomodulatory activity of pectins, which was particularly significant for highly branched polymers [10]. Some pectin fractions demonstrate free radical scavenging activity [11]. Certain chemical modifications, for example, alkali treatment and heat-controlled enzymatic treatment, contribute to anti-tumorigenic actions of pectins, which are stronger for pectins with lower molecular weight and higher RG-I to HG ratios [12,13]. The results of pilot clinical trials indicate that modified citrus pectin of molecular weights lower than 15 kDa and a degree of esterification under 5%, can be absorbed from the small intestinal epithelium and reach circulation, where they inhibit the development of secondary tumors in prostate cancer patients [14]. It is believed that galectin-3 (gal-3) inhibition is responsible for the anti-cancer actions of low molecular weight pectins. Galectin-3 is a protein that specifically binds β-galactosides and is able to modulate functions important for cell survival, migration and metastasis [15].

According to our knowledge, we are the first to propose the application of monocatalytic enzyme preparations for pectin isolation. The results of these studies demonstrated that the use of endo-cellulase or endo-xylanase for pectin extraction leads to comparable and often higher yield as compared to acid process. The enzyme released polymer has significantly larger molecular weight and contains more galacturonic acid and neutral sugars [16]. According to the literature, such parameters are associated with improved gelifying properties [2,4]. We hypothesized that the enzyme isolated pectins should also show better radical-scavenging and anti-cancer activities, due to their composition, namely, high content of galactose, rhamnose and phenolic compounds. So far, a considerable anti-cancer potential has been confirmed only for low molecular weight pectin fractions [13,14]. In order to test our hypothesis, we chose human adenocarcinoma HT-29 and murine malignant melanoma B16F10 cell lines, as in vitro models for tissues that could possibly come into contact with such polymers in natural conditions (i.e., present in gastrointestinal tract or applied directly onto skin surface).

## 2. Results and Discussion

### 2.1. The Content of Ferulic Acid (FA) and Total Phenols

The total phenolic content of compounds in apple pomace depends on multiple factors (i.e., apple cultivars, juice pressing method and the conditions of pomace drying), and varies considerably in literature. Some studies report values as high as 10% of phenols in pomace [17], in some others the phenolic content is estimated at 0.15–0.85% of dry weight [18,19]. The content of phenolics in the pomace used in our experiments was 0.22% (calculated as ferulic acid equivalent), so it falls within the latter range (Table 1).

The data indicate that between 27 and 42% of these phenolic compounds were bound to the isolated pectins so tightly that they were co-eluted and subsequently co-precipitated with them. Within these phenolics, ferulic acid made up to 6.3–16.44%. The phenolic content was significantly higher in the enzymatically isolated polysaccharides which were subjected to much milder conditions of pH and temperature (pH 5.0, 40 °C). One gram of such a pectin preparation contained 79–930 µg of total phenolic compounds, including 63.5–152.8 µg of ferulic acid. For comparison, 1 g of acid extracted (pH 2.0, 85 °C) pectin preparation contained only 590 µg of total phenols, including 37.3 µg of FA.

Our studies did not provide an unequivocal answer to the question of whether FA and other accompanying phenolic compounds were physically associated with pectins, or whether they were components of small fractions of hemicelluloses, which were released by the applied enzymes together with pectins and subsequently co-precipitated. It is known from the literature that ferulic acid may be esterified to the O-2 position of the arabinose residues in the α-(1,5)-linked-arabinan backbone, but may also be bound to the O-5 on the terminal arabinose, or, to a much lesser extent, at the O-6 position of the galactopyranosyl residues in the β-(1,4)-galactan chains [20]. Arabinan and galactan chains are natural components of the RG-I pectin fraction. FA is also present in xyloglucans, which are covalently linked to RG-I by glycosidic bonds or diferulic bridges [21]. The statistical analysis of the correlation between the contents of galactose (Gal), arabinose (Ara), glucose (Glu) and xylose (Xyl) and the ferulic acid content in the polysaccharides obtained in our experiments, delivered the regression coefficient values of: R^2^ = 0.558 with *p* = 0.0001 (for Gal), R^2^ = 0.312 with *p* = 0.018 (for Ara), R^2^ = 0.196 with *p* = 0.021 (for Glu) and R^2^ = 0.169 with *p* = 0.017 (for Xyl). Therefore, only in the cases of Gal and Ara, can the correlation be regarded as high and moderate, respectively. These results suggest that FA, present in our polysaccharide preparations, was most probably a natural RG-I component and not a “contamination” from xyloglucan.

The presence of phenols in pectin of various sources was discussed previously by other researchers [22]. For example, sugar beet pectin is regarded as particularly rich in phenols, as it contains up to 0.7% (calculated as ferulic acid equivalents), when extracted at high pH [23], and a ferulation degree (DF) that oscillates around 0.27% [24]. In orange peel pectins, FA content is estimated at 160 µg/g for acid extraction and up to 740 µg/g for water extraction [25]. To our knowledge, it has never been shown before that FA is a natural component of apple pectin and that its content may vary across a wide range, depending on extraction conditions such as pH, but also the type of enzyme used during the extraction process. As shown in Table 1, the highest level of FA was present in the pectin preparation isolated with endo-xylanase. Only less than half of this amount was assessed in the pectin isolated with endo-cellulase. These differences in FA content can only be attributed to the enzyme type, since pH, temperature and reaction times were the same in both cases. Apparently, endo-xylanase was able to liberate homogalacturonic acid from the cell-wall structures together with arabinans and galactans that constitute RG-I. On the contrary, these fragments could be degraded relatively easily in acidic conditions.

### 2.2. In Vitro Antioxidant Activity of Pectin

**Figure 1:**
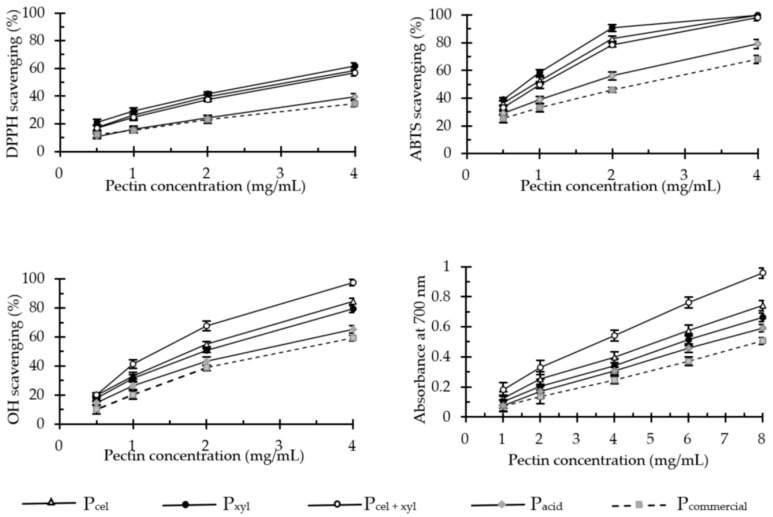
DPPH^•^, ABTS^•+^ and ^•^OH scavenging activity and ferric-reducing power of apple pectins (in vitro tests). Data are presented as the mean ± SD of four independent experiments. P_cel_—cellulase-extracted pectin; P_xyl_—xylanase-extracted pectin; P_cel + xyl_—pectin extracted with both cellulase and xylanase; P_commercial_—commercial apple pectin.

The antioxidant activity of pectins was determined with widely used methods that employ synthetic stable free radicals ABTS^•+^ and DPPH^•^, as well as hydroxyl radicals generated in the Fenton reaction. We also assessed the pectin’s ability to reduce ferric ions in vitro. Each method unequivocally demonstrated that antioxidant activities of pectins vary significantly depending on the isolation method (Figure 1), but it always remains about 200 times lower in comparison to the well-established anti-oxidative compounds, such as pure ferulic acid or Trolox.

Conventionally acid-extracted pectins (P_acid_ and P_commercial_) were significantly less effective in free radical scavenging and the reduction of ferric ions than those which were enzymatically extracted (P_cel_, P_xyl_ and P_cel + xyl_). As it is shown in Table 2, in extreme cases, the effective doses (IC_50_) of acid-extracted pectins were three times higher than in enzyme-isolated pectins.

Comparable and relatively low antioxidant activity of commercial pectin preparations was previously reported by Dou et al. [26]. Additionally, Wang et al. [11] demonstrated that free radical scavenging properties of apple pectin could be enhanced by use of subcritical water at high temperatures (130–170 °C) during the extraction. Depending on the extraction temperature, the IC_50_ values of such pectins oscillated between 1.4–3.5 mg/mL for DPPH^•^ scavenging and about 1 mg/mL for ABTS^•+^ [11]. Our results proved that pectins extracted enzymatically from apple pomace had a similarly high potential for the neutralization of both DPPH^•^ (IC_50_ = 2.75–3.26 mg/mL) and ABTS^•+^ (IC_50_ = 0.76–1.58 mg/mL). However, their molecular mass was up to 30 times higher than that of pectins isolated with the use of subcritical water. Importantly, both methods of antioxidant activity assessment produced very high positive linear correlation (R^2^ = 0.98, *p* = 0.001).

As we mentioned earlier, the replacement of the acid with the enzyme preparations enabled us to obtain the apple pectins, which not only scavenged the synthetic free radicals more efficiently, but also naturally occurring hydroxyl radicals (IC_50_ = 1.18–2.00 mg/mL). We observed a linear correlation of this activity (the equation: y = 0.3945 × −0.2138; R^2^ = 0.99 with *p* = 0.000) with the pectin’s reducing power (Table 2). This tight correlation between the polysaccharide’s ability to scavenge ^•^OH and ferric ions reduction was also postulated in the works by You et al. [27] and Dou et al. [26]. Nevertheless, these authors did not analyze the type and significance of this correlation. The relation between these two parameters seems quite obvious, because hydroxyl radicals are generated in the reactions of transition metals, in other words, in the Fenton reactions. Transition metal ions are easily chelated by pectins [8].

In search of the factors responsible for the significantly higher antioxidant activity of enzyme-isolated pectins as compared to acid-extracted pectins, we tested the force of the linear correlation between IC_50_ values and the following parameters: molecular weight (M_w_), degree of methylation (DM) and the galacturonic acid content (GalA), total phenols, FA, proteins, glucose, galactose, arabinose, rhamnose, xylose, fucose and mannose. The relationships with the highest regression coefficients are presented in Table 3.

The results indicate that the pectin activity against DPPH^•^ and ABTS^•+^ increased proportionally to the content of phenols (R^2^ = 0.94–0.97) and fucose (R^2^ = 0.89–0.93). A positive, but less significant correlation was observed for the protein content (R^2^ = 0.80–0.86) and the degree of methylation (R^2^ = 0.64–0.70), as well as for the FA and rhamnose content. The ^•^OH scavenging activity and reducing power showed a different pattern: these two parameters increased mainly with high rhamnose (R^2^ = 0.77–0.83) content, as well as with fucose (R^2^ = 0.53–0.59) and phenolic compounds content (R^2^ = 0.52–0.58). The impact of protein content and DM on the ^•^OH scavenging and Fe(III) reducing properties was negligible. The presented results are supported by the literature data. Several studies have postulated that the presence of protein or peptide moiety in polysaccharides isolated from plant matrices is partially responsible for the radical scavenging effect [28]. Furthermore, phenolic compounds, especially phenolic acids, can play an important role in the overall radical scavenging ability of the same polysaccharides, for example, xylans and xylooligosaccharides [29]. Smirnov et al. [22] even suggest that a very high DPPH^•^ and ^•^OH scavenging activity of their pectin preparations resulted from a high content of phenolic compounds (more precisely: the compounds that reacted with Folin–Ciocalteu reagent) and protein. Ferulic acid in the effective doses of our pectin preparations (IC_50_, RP_0.5_) constituted between 0.06 and 0.89 µg. Assuming that pectin-bound FA was as equally active as its free form (Table 2), it was responsible for maximally 3.7% of the free radical-scavenging effect of these doses.

It was also proved that the compositions and ratios of individual monosaccharides, as well as types of glycosyl linkages, would be of concern in modulating the antioxidant properties of polysaccharides [30]. Especially, rhamnose and mannose showed high positive coefficients in linear regression analysis [31]. The antioxidant potential of sugars also depends on their polymerization degree, as well as the degree of methylation in cases of pectins. It was suggested that polysaccharides with low molecular weight would possess more terminal reductive hydroxyl groups (per unit mass) to accept and eliminate free radicals. Therefore, a physical, chemical or enzymatical degradation of polysaccharides usually significantly improves their antioxidant activity [32]. Additionally, in case of pectins the low methylation degree facilitates metal ion chelation, which can translate into inhibition of the Fenton reactions. However, our results indicate that pectins with lower molecular weights and lower degrees of methylation were significantly less effective in scavenging of free radicals than the high molecular weight preparations with higher degrees of esterification (DE). We need to bear in mind that molecular weight was not the only variable and these polymers were obviously not identical. The applied extraction method has a strong impact on the composition of the obtained pectin preparation. The enzymatically isolated pectins contained significantly more rhamnose, fucose, protein and phenolic compounds (Table 1 and Table 5). This is probably the reason for their three-fold higher antioxidant potential, as compared to conventional pectin preparations, despite considerably higher molecular weight and higher DE. In conclusion, the antioxidant activity of pectin depends strongly on the amount and type of neutral sugars (NS) as well as on protein and phenolic contaminants, and only to lesser extent on the molecular weight and the degree of methylation.

### 2.3. Anti-Cancer Activity of Pectin Preparations

Dietary pectins are digested only by microbiota present in the last section of the gastrointestinal tract, therefore in their natural form they cannot reach circulation. However, a direct contact between pectins with stomach and intestinal mucosa is possible and likely. Such an interaction exerts health promoting anti-inflammatory and immunomodulating effects on epithelial cells [33]. In a similar fashion, pectins applied topically can come into physical contact with skin epidermal cells. Therefore, we decided to find out if high molecular weight pectins exert any beneficial biological effects that could be a therapeutic support against cutaneous and gastric malignancies. Commercial pectin preparations, including apple pectins, do not exert any particular anti-cancer actions [15]. On the other hand, pectins extracted from various materials and subsequently chemically or enzymatically modified to low molecular weight, highly branched fractions, rich in RG-I, have been shown to decrease proliferation rate and migration, as well as induce apoptosis in a variety of cancer cell lines [13,15]. Commercial enzyme-modified pectins are currently being tested in clinical trials recruiting patients with neoplastic diseases, for example, PectaSol-C for the treatment of prostate cancer [14].

We tested the biological activity of apple pectins extracted with a conventional acid treatment or enzymatically. Notably, the latter method increases yield, at the same time remaining environmentally friendly. All the pectin preparations with high molecular weights, contained 60–75% highly methylated (DM = 56–73%) polygalacturonate, but differed significantly in their neutral monosaccharide profiles and their protein and phenolics content (Table 1 and Table 5). Enzyme-extracted pectins were richer in rhamnose, galactose, fucose and FA than acid-extracted preparations, which probably indicates that the proportion of RG-I in their structure was significantly larger. In vitro tests revealed that such pectins exerted no adverse effects on normal fibroblast L929 cells (Table 4).

**Figure 2:**
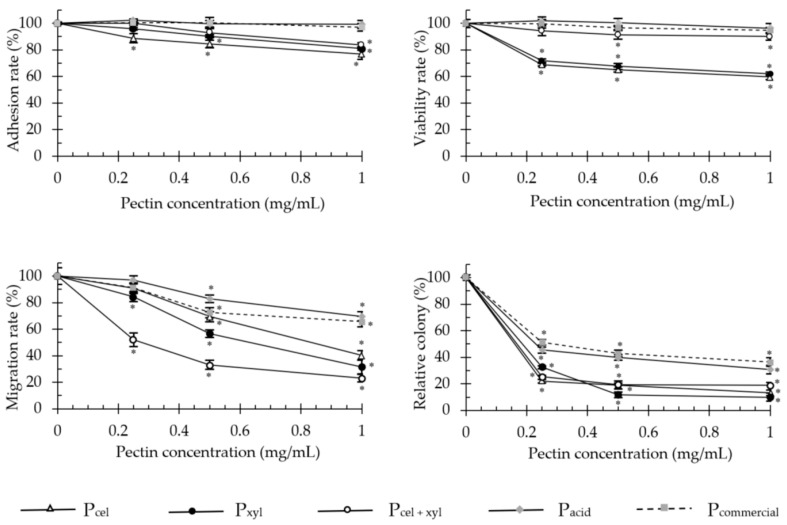
The effect of apple pectins on HT-29 cell proliferation, migration, adhesion and growth in soft agar in vitro. The values represent the means ± SD of cells/colonies (% control). All the experiments were performed thrice in triplicate. * *p* ˂ 0.001 vs. the control group (Tukey test). P_cel_—cellulase-extracted pectin; P_xyl_—xylanase-extracted pectin; P_cel + xyl_—pectin extracted with both cellulase and xylanase; P_commercial_—commercial apple pectin.

**Figure 3:**
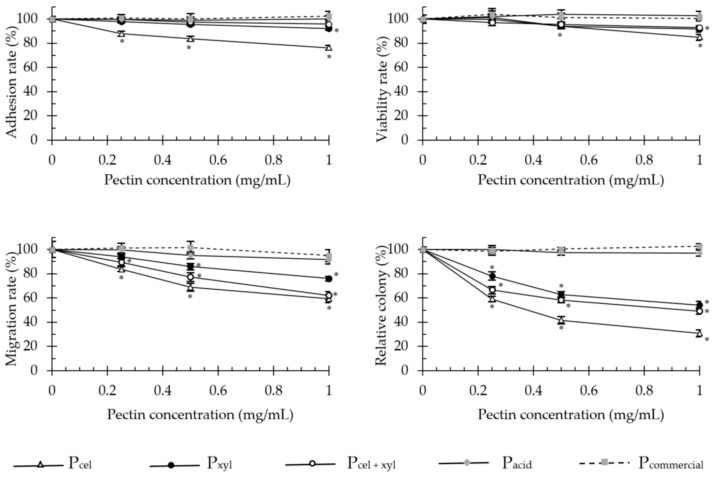
The effect of apple pectins on B16F10 cell proliferation, migration, adhesion and growth in soft agar in vitro. The values represent the means ± SD of cells/colonies (% control). All the experiments were performed thrice in triplicate. * *p* ˂ 0.001 vs. the control group (Tukey test). P_cel_—cellulase-extracted pectin; P_xyl_—xylanase-extracted pectin; P_cel + xyl_—pectin extracted with both cellulase and xylanase; P_commercial_—commercial apple pectin.

Enzymatically generated pectins significantly decreased cell adhesion, proliferation, invasion and anchorage-independent growth of adenocarcinoma HT-29 (Figure 2) and malignant melanoma B16F10 (Figure 3) cells. The colorectal cancer cells were much more sensitive to the pectin treatment than melanoma cells.

The enzymatically extracted pectins at the concentration of 1 mg/mL, decreased the proliferative capacity of HT-29 cells (after 48 h incubation) and invasion through extracellular matrix Martgel layer (24 h incubation) of up to 40.4 and 76.9%, respectively. Soft agar colony formation was diminished by up to 90%. For melanoma cells these values dropped to 15%, 40.6% and 69.3%, respectively. Only in the adhesion assays did both cell types exhibit similar sensitivity to the presence of pectins and responded with 23–24% decrease in adherence, at maximum. In the literature, modified sugar beet pectin (1 mg/mL, for 48 h) reduced the number of viable HT-29 cells by 20.7% [15], and low molecular weight apple and citrus pectins extracted by subcritical water, reduced the viable cell number by 19 and 17%, respectively [11]. In turn, Vayssade et al. [34] demonstrated that RG-I fraction of okra pectin administered in 1 mg/mL dose for 48 h, inhibited B16F10 cell proliferation by 39% in conventional 2D culture and by 75% in aggregates on anti-adhesive substratum (3D). These data show a trend similar to our results obtained for enzymatically extracted unmodified apple pectins.

Both studied cell lines have been shown to overexpress β-galactoside binding protein (gal-3) [35,36]. Gal-3 is involved in tumor development, mainly through: (i) facilitation of tumor cell adaptation for survival in stressed conditions; (ii) induction of tumor cell detachment and migration upon secretion; and (iii) attraction of monocytes and endothelial cells into a tumor mass, inducing both directly and indirectly the process of angiogenesis [37]. The intensity of pectin anti-cancer action has been directly linked with the gal-3 expression level on the surface of cancer cells [38]. The galactose residues in pectin are similar to many tumor-associated antigens (TAAs) that are responsible for interaction with gal-3. However, in contrast to TAAs, their binding to gal-3 exerts antiproliferative effects and therefore could be one of the possible mechanisms through which pectins inhibit tumorigenesis [12,38]. This notion is in agreement with our data, where the regression analysis demonstrated a highly significant positive correlation between the galactose content and anti-cancer activity (R^2^ = 0.58, *p* = 0.000 for B16F10 cell adhesion; R^2^ = 0.95, *p* = 0.000 for anchorage-independent growth of HT-29 cells). We also noticed that the strength of pectin anti-cancer activity significantly correlated with the high phenols content. In this case, R^2^ coefficient fell within the range of 0.54–0.78 for HT-29 cells and 0.60–0.94 for B16F10 cells. Such a correlation has never been mentioned in the literature before, but it is not unexpected. It is well established that both simple and complex phenolic compounds, even in low doses (0.2 µg/mL for FA and 100 µg/mL for anthocyanins), exert potent pleiotropic anti-cancer effects [39,40]. Unfortunately, the doses exceeding 0.4 mg/mL are frequently cytotoxic for healthy nontransformed cells [40]. The content of phenolic compounds in the highest pectin dose used in this study (1 mg/mL) varied between 0.38 and 0.93 µg, including 0.037–0.15 µg of FA. It was therefore a sufficiently high amount to fortify the anti-cancer potential of pectins and simultaneously low enough to avoid affecting the viability of healthy cells.

Pectins obtained from various sources have been shown to exert health promoting through their anti-oxidative capacity [33]. The studies on various bioactive phytochemicals pointed out the relationship between the anti-oxidative potential and anti-cancer properties [41,42]. It might be interpreted in the context of the mutagenic activity of reactive oxygen species (ROS), as well as to ROS-mediated activation of protooncogenes and inactivation of tumor suppressor genes [43,44]. Cancer cells have elevated intracellular ROS levels, due to the impairment of cytoprotective mechanisms against oxidative stress, which is regarded as a cancer driving force [43]. Additionally, the extracellular ROS, such as hydroxyl radical and superoxide, are present in the tumor surrounding tissue, as they are released by activated macrophages and neutrophils during the immune response called a respiratory burst. The inflammation at the tumor site also leads to elevated ROS and reactive nitrogen species (RNS) production and induces polarization of tumor-associated macrophages towards the M2 phenotype. The M2 macrophages create tumor-permissive conditions though the release of particular cytokine profiles (IL-4, IL-13, IL-10), growth factors (epidermal growth factor, EGF) and proteases that cleave extracellular matrix proteins and support cancer expansion [45]. The secreted cytokines induce vasodilation and increased vascular permeability, which further facilitate tumor invasion and metastasis. Therefore, the anti-oxidative activities of pectins observed in our experiments may help to resolve inflammation and counteract the cancer-permissive modulation of the tumor environment.

In conclusion, the recent studies on anti-cancer properties of pectins were mostly focused on the gal-3 directed inhibitory action of modified low molecular weight pectins. It was suggested that due to limited solubility of native apple and citrus pectins in water, they are unable to interact with gal-3 and act as antagonists [38]. Our results indicate that the apple pectins in their native forms, isolated with xylanase and cellulase, efficiently inhibit proliferation and invasion of cancer cells. Their anti-cancer potential can be attributed not only to the presence of galactose residues, which can inhibit gal-3, but also to a high phenolics content. Importantly, such pectin preparations are well-tolerated by normal healthy cells with no signs of cytotoxicity. Our results showing the significant anti-cancer potential were obtained from in vitro experiments, which is a serious limitation. The real effect of the pectin application should be tested in the animal models, as well.

## 3. Materials and Methods

### 3.1. Chemicals and Reagents

DPPH, ABTS, EDTA, ferulic acid, Trolox, thiobarbituric acid (TBA), deoxyribose, ascorbic acid, ferric chloride, potassium ferricyanide and trichloroacetate were from Sigma/Aldrich Chemical Co., Germany; hydrogen peroxide, NaOH, HCl, ethyl acetate, acetonitrile, methanol, acetic acid and formaldehyde were from POCH, Avantor Performance Materials, Poland; agarose, crystal violet and Coomassie Brilliant Blue G250 were purchased from Merc KGaH, Germany; RPMI 1640 medium and fetal bovine serum (FBS) were from Gibco; glutamine, streptomycin, penicillin, amphotericin B, bovine serum albumin (BSA) and Ca/Mg-containing phosphate buffered saline (PBS) were from Pan Biotech, Germany; sodium pyruvate was from Corning, USA; BD BioCoat Matrigel Invasion Chambers were purchased from BD Bioscience, USA.

### 3.2. Pectin

Pectins were isolated from dried apple pomace using sulfuric acid (pH 2.0, 85 °C, 3 h) or enzymatic preparations endo-β-1,4-xylanase (EC 3.2.1.8) from *Trichoderma viride* (Sigma/Aldrich Chemical Co., Germany, Cat. No. X3876) and endo-cellulase (endo-β-1,4-glucanase, EC 3.2.1.4) from *Trichoderma viride* (Sigma/Aldrich Chemical Co., Germany, Cat. No. C9422). Both enzymes were used at doses of 50 U/1 g of pomace, at pH 5.0, in 40 °C, for 10 h, according to a method described by Wikiera et al. [16]. The enzymatic preparations did not exhibit any additional off target activities [16].

Commercial apple pectin (P_commercial_) isolated with sulfuric acid obtained from Pektowin (Poland) was used as a reference. The primary chemical composition of pectin preparations used in the experiments is presented in the Table 5A,B.

### 3.3. Ferulic Acid and Total Phenols Determination

The content of phenols was assessed according to Machu et al. [46], with some modifications. Pectin samples (200 mg) were saponified in 10 mL of 1 M NaOH at 37 °C for 24 h, then centrifuged (4000 rpm, 10 min, MPW 352R Med. Instruments, Warszawa, Poland) and the supernatants were acidified to pH 3 with 2 M HCl. The phenolic compounds were extracted into ethyl acetate (4 × 5 mL) and centrifuged (4000 rpm for 10 min). The pooled fractions were evaporated to dryness at 40 °C and the residue was dissolved in 10 mL of 50% methanol and membrane-filtered (0.22 µm, Sigma/Aldrich Chemical Co, Steinheim, Germany). Ten microliters of this solution were injected into an UltiMate 3000 HPLC system (Dionex, Sunnyvale, CA, USA) equipped with a C18 column Gemini-NX 150 × 4.6 mm (Phenomenex, Torrance, CA, USA) and a DAD detector. The elution was carried out with a two-component mobile phase: (A) water + acetic acid (99:1) and (B) water + acetonitrile + acetic acid (67:32:1). The gradient profile was 0–10 min, 10–20% B; 10–16 min, 20–40% B; 16–20 min, 40–50% B; 20–25 min, 50–70% B; 30–40 min, 70–10% B; 40–45 min 10% B with a flow rate of 1 mL/min, at constant temperature of 23 °C. The detection was performed at 275 nm (UV detector, Dionex, Sunnyvale, CA, USA). Chromatographic peak of ferulic acid was identified by a comparison of retention time with the pure standard. The content of ferulic acid and total phenols (ferulic acid equivalents) was expressed in μg/g of dry mass of a sample.

### 3.4. Measurement of Antioxidant Activities In Vitro

#### 3.4.1. DPPH Radical Scavenging Activity

The DPPH radical scavenging assay was carried out according to Rha et al. [47] with some modifications. One milliliter of the pectin solution in water (0.5–4 mg/mL) was mixed with 1 mL of freshly prepared DPPH^•^ in methanol (0.2 mM) and the absorbance at 516 nm (Specord 40, Analytik Jena, Jena, Germany) was measured after 15 min incubation at room temperature in the dark. Pure ferulic acid and Trolox were used as references. The capability to scavenge DPPH radical was expressed as IC_50_, in other words, the pectin concentration (mg/mL) required for 50% scavenging of DPPH^•^ in the reaction conditions.

#### 3.4.2. ABTS^•+^ Scavenging Activity

The ABTS radical scavenging assay was carried out according to Braca et al. [48] with some modifications. One milliliter ABTS^•+^ solution in phosphate buffer (0.4 M, pH 7.5), whose absorbance at 734 nm was adjusted to 0.7, was mixed with 1 mL of pectin solution in phosphate buffer (0.5–4 mg/mL). The absorbance of the mixture was measured at 734 nm (Specord 40, Analytik Jena, Germany) after 6 min incubation at room temperature in the dark. Pure ferulic acid and Trolox were used as references. The capability to scavenge ABTS^•+^ was expressed as IC_50_, in other words, the pectin concentration (mg/mL) required for 50% scavenging of ABTS^•+^ in the reaction conditions.

#### 3.4.3. Hydroxyl Radical Scavenging Activity

The ability of the pectins to scavenge ^•^OH was determined using deoxyribose assay, originally described by Marambe et al. [49] with some adaptations. The pectin solutions (0.5–4 mg/mL) were prepared in potassium phosphate buffer (20 mM, pH 7.4). The assay mixture contained: 563 µL of pectin solution, 20 µL 0.5 mM FeCl_3_, 21 µL 2.4 mM EDTA, 70 µL 20 mM deoxyribose and 10 µL 5 mM ascorbic acid. Finally, 71 µL 1 mM H_2_O_2_ was added and the whole mixture was incubated at 37 °C for 1 h. Thereafter, 750 µL of 1% (*w/v*) thiobarbituric acid (TBA) and 750 µL of 2.8% (*w/v*) trichloroacetate (TCA) were added and the tube was incubated at 100 °C for 20 min. After cooling to room temperature, the absorbance was measured at 532 nm (Specord 40, Analytik Jena, Germany) against a reference sample (in which TBA and TCA were added prior to H_2_O_2_ solution and incubation at 37 °C). A control reaction with buffer instead of pectin was also performed. Pure ferulic acid and Trolox were used as references. The ^•^OH scavenging activity was expressed as IC_50_, in other words, the pectin concentration (mg/mL) required for 50% scavenging of ^•^OH generated in the reaction conditions.

#### 3.4.4. Determination of Reducing Power

The reducing power of pectins was determined according to the method of Ardestani and Yazdanparast [50]. Pectin samples (1 mL) with concentrations of 1–8 mg/mL in 0.2 M phosphate buffer (pH 6.6) were added to 2.5 mL of 1% (*w/v*) potassium ferricyanide. The mixture was incubated for 20 min at 50 °C, and then 2.5 mL of 10% (*w/v*) TCA was added to the reaction mixture and centrifuged at 3000 rpm for 10 min (MPW 352R Med. Instruments, Poland). The supernatant (2.5 mL) was mixed with 2.5 mL of double distilled water and 0.5 mL of 0.1% (*w/v*) ferric chloride. After 10 min of reaction, the absorbance was measured at 700 nm (Specord 40, Analytik Jena, Germany). Reducing power was expressed as RP_0.5_ defined as a sample concentration (mg/mL) that produces 0.5 absorbance unit at 700 nm. Pure ferulic acid and Trolox were used as references.

### 3.5. Evaluation of Pectin Anti-Cancer Activities In Vitro

#### 3.5.1. Cell Lines and Culture

HT-29 (human colorectal adenocarcinoma, ATCC #HTB-38), B16F10 (mouse skin melanoma, ATCC #CRL-6475) and L929 (non-tumorogenic mouse fibroblasts, ATCC #CCL-1) cell lines (purchased from American Type Culture Collection), were grown in RPMI 1640 medium, supplemented with 10% fetal bovine serum, 2 mM glutamine, 100 µg/mL streptomycin, 100 U/mL penicillin and amphotericin B at 37 °C in a humidified atmosphere of 5% CO_2_ (Model 3100, Thermo Electron Co, Waltham, MA, USA).

#### 3.5.2. Cell Adhesion Assays

HT-29, B16F10 and L929 cells grown on a 24-well tissue culture plates (1 × 105 cells per well) were incubated for 18 h in the standard growth media supplemented with 0 (control), 0.25, 0.5 and 1 mg/mL pectin. After that, the media were aspirated and the plates were rinsed twice in Ca/Mg-containing phosphate buffered saline (PBS) and rotated at 250 rpm for 2 h on an orbital shaker. The cells released into the buffer were collected and pelleted by centrifugation. Both fractions of cells: those released, and those adherent, were fixed with a buffered formaldehyde solution (3.7%, pH 7.4) for 15 min and stained in 0.5% crystal violet solution in 50% methanol for 10 min. After removal of the staining solution, the cells were washed with water and destained for 30 min in the 50 mM citrate buffer in 50% methanol to discharge the dye from the cells. The absorbance (at 540 nm) of the supernatant samples was measured in a microplate reader (Bio-Rad 680, Bio-Rad Laboratories Inc, Pleasanton, CA, USA). The percentage of cells remaining on the plate after rotation and representing relative cell adhesion strength, was calculated in each case.

#### 3.5.3. Cell Proliferation Assays

The effect of crude pectin preparations on HT-29, B16F10 and L929 cell proliferation was assessed by crystal violet staining. Briefly, the cells (2 × 104 cells per well) were seeded in 24-well plates in 0.5 mL of the cell culture medium and left to adhere for 18 h. Then, the cells were treated for 48 h with the pectin solutions in various concentrations (0.25, 0.5 and 1 mg/mL), prepared in the cell culture medium. After 48-h treatment the cells were washed in PBS, fixed with formaldehyde, stained with crystal violet and the absorbance was measured at 540 nm as described above in the adhesion assays. The results from the pectin treated samples were expressed as percent of control.

#### 3.5.4. Anchorage-Independent Growth Assay (Colony Formation in Soft Agar)

In order to determine cell growth in anchorage-independent conditions, HT-29 and B16F10 cells were suspended in 0.4% agarose/RPMI with 10% FBS, penicillin, streptomycin, 1 mmol/L sodium pyruvate and the pectin solutions of varying concentrations (0.25, 0.5 and 1 mg/mL) and plated onto 0.8% agarose/RPMI–covered 6-well plates. The plates were kept in the cell culture incubator for 10 d, whereupon the colonies with diameters bigger than 50 µm were counted under a light microscope (AxioVert Z1 inverted fluorescent microscope, Zeiss, Göttingen, Germany) with Quantity One software (Bio-Rad, USA).

#### 3.5.5. Cells Migration by Transwell Assay (Invasion)

Invasion of HT-29 and B16F10 cells was determined using BD BioCoat Matrigel Invasion Chambers with the porous polycarbonate inserts (pore size 8.0 µm, Sigma/Aldrich Chemical Co, Darmstadt, Germany) coated with Matrigel. The lower compartment was filled with the regular serum containing cell culture medium. HT-29 and B16F10 cells (5 × 104 cells per insert) were suspended in 300 µL of serum-free medium (SFM, RPMI, 2 mM glutamine, antibiotics and antimycotics as in the regular growth medium, 1% bovine serum albumin), placed in the upper chambers and left to attach for 24 h. After that, the medium in the upper chambers was changed to SFM with varying pectin concentrations (0.25, 0.5, 1 mg/mL) and left for the next 24 h. The cells from the upper surface of inserts were carefully removed with cotton swabs and the bottom surfaces (containing the migratory cells) were fixed and stained in Coomassie Brilliant Blue G250 solution for 30 min, rinsed and counted in the light microscope (Techno Meiji 5400, Saitama, Japan).

### 3.6. Statistical Analysis

The statistical significance of differences among the means was evaluated with ANOVA, Fisher’s least significant difference and Tukey multiple-range post hoc tests at significance levels of 0.05 and 0.001 using Statistica Software for Windows, version 13 (StatSoft Inc., Poland). The correlations were tested using multiple regression (Pearson’s test).

## 4. Conclusions

Monocatalytic endo-cellulase and endo-xylanase preparations are able to effectively release pectins from apple pomace. The pectin preparations are characterized by high molecular weight (419–899 kDa) and large GalA content (61.1–74.7%). These pectins possess a highly superior radical scavenging activity (twice as high on average) as compared to acid extracted pectins. As opposed to the latter, the enzymatically isolated pectins potently inhibit proliferation, adhesion and invasion of human adenocarcinoma HT-29 and murine melanoma B16F10 cells, while remaining neutral to nontransformed cells. The antioxidative potential of these pectins correlates positively with the content of phenolic compounds, including FA, but also with the Fuc and Rha content in their structure. The Rha content seems to be especially important for ^•^OH scavenging and ferric ions reduction. The high content of galactose and phenolic compounds enhances the anti-cancer activities of pectins. To sum up, our results indicate that on one hand the enzymatic extraction is an efficient method of pectin release from plant cell wall structures, and on the other hand it simultaneously allows retaining of a variety of health-promoting activities in the pectin polymer. In perspective, the detailed molecular mechanisms that lie behind the anti-proliferative, anti-adhesion and anti-invasive action of enzymatically extracted pectins should be characterized in order to fully exploit their therapeutic potential.

## Figures and Tables

**Table 1:** The content of ferulic acid (FA) and total phenolic compounds in apple pectin and apple pomace.

Pectin		Total Phenols Area (mAU × min)	FA Content (µg/g)	Total Phenols Expressed in FA (µg/g)
	Pomace			
P_cel_		1.244 ± 0.03 ^c^	63.56 ± 0.59 ^c^	840 ± 1.42 ^d^
P_xyl_		1.546 ± 0.04 ^e^	152.87 ± 0.84 ^e^	930 ± 1.08 ^e^
P_cel + xyl_		1.301 ± 0.08 ^d^	122.38 ± 0.51 ^d^	790 ± 0.91 ^c^
P_acid_		0.949 ± 0.14 ^b^	37.31 ± 0.39 ^b^	590 ± 1.13 ^b^
P_commercial_		0.592 ± 0.03 ^a^	24.86 ± 0.41 ^a^	380 ± 0.59 ^a^
Apple pomace		4.009 ± 0.19 ^f^	220.04 ± 1.51 ^f^	2220 ± 2.07 ^f^

Data are presented as the mean ± SD of four independent experiments, different letters in the same column indicate the statistical significance (*p* < 0.05, LSD test). P_cel_—endo-cellulase-extracted pectin; P_xyl_—endo-xylanase-extracted pectin; P_cel + xyl_—pectin extracted with both endo-cellulase and endo-xylanase; P_commercial_—commercial apple pectin.

**Table 2:** Antioxidant activity of acid and enzymatically extracted apple pectins.

Pectin		IC_50_ (mg/mL)			RP_0.5_ (mg/mL)
	References	DPPH^•^	ABTS^•+^	^•^OH	
P_cel_		3.02 ± 0.07 ^d^	0.92 ± 0.06 ^d^	1.87 ± 0.05 ^d^	5.14 ± 0.08 ^d^
P_xyl_		2.75 ± 0.09 ^c^	0.76 ± 0.05 ^c^	2.00 ± 0.07 ^e^	5.91 ± 0.07 ^e^
P_cel + xyl_		3.26 ± 0.08 ^e^	1.14 ± 0.08 ^e^	1.18 ± 0.07 ^c^	3.49 ± 0.09 ^c^
P_acid_		5.24 ± 0.08 ^f^	1.58 ± 0.09 ^f^	2.37 ± 0.05 ^f^	6.56 ± 0.10 ^f^
P_commercial_		9.01 ± 0.11 ^g^	2.45 ± 0.08 ^g^	2.98 ± 0.06 ^g^	8.00 ± 0.11 ^g^
			(µg/mL)		(µg/mL)
FA		8.39 ± 0.14 ^b^	4.02 ± 0.09 ^b^	4.67 ± 0.10 ^b^	12.04 ± 0.21 ^b^
Trolox		5.14 ± 0.12 ^a^	8.82 ± 0.17 ^a^	3.51 ± 0.08 ^a^	5.43 ± 0.19 ^a^

Data are presented as the mean ± SD of four independent experiments, different letters in the same column indicate the statistical significance (*p* < 0.05, LSD test). IC_50_—the amount of pectin (g/mL) that causes 50% scavenging of free radicals; RP_0.5_—reducing power was defined as pectin sample concentration (mg/mL) that produces 0.5 absorbance unit at 700 nm.

**Table 3:** Multiple regression analysis for IC_50_ and RP_0.5_ antioxidant capacity with degree of methylation (DM) and molecular weight (M_w_) of pectins, and the contents of: ferulic acid (FA), phenols, protein, rhamnose, fucose and mannose.

Variable		Parameter Estimate	Standard Error	R^2^	*p* Value
Dependent	Independent				
DPPH^•^	M_w_	2.0081	1.2200	0.3438	0.0065
	DM	1.4861	3.2304	0.6406	0.0000
	FA	1.6343	0.6960	0.5654	0.0001
	Phenols	0.5931	0.4923	0.9427	0.0000
	Protein	1.1036	0.5558	0.8018	0.0000
	Rhamnose	1.6060	2.0237	0.5803	0.0000
	Mannose	1.9040	0.5848	0.4101	0.0023
	Fucose	0.8159	0.5132	0.8917	0.0000
ABTS^•+^	M_w_	0.4685	0.2848	0.4673	0.0008
	DM	0.3503	0.7625	0.7022	0.0000
	FA	0.4140	0.1763	0.5839	0.0000
	Phenols	0.1113	0.0902	0.9698	0.0000
	Protein	0.2353	0.1185	0.8656	0.0000
	Rhamnose	0.4463	0.5623	0.5167	0.0003
	Mannose	0.4695	0.1442	0.4651	0.0009
	Fucose	0.1684	0.1059	0.9311	0.0000
^•^OH	M_w_	0.6172	0.3750	0.0474	0.3562
	DM	0.4740	1.0317	0.4382	0.0014
	FA	0.4541	0.1934	0.4843	0.0006
	Phenols	0.4103	0.3406	0.5792	0.0000
	Protein	0.4687	0.2360	0.4506	0.0011
	Rhamnose	0.2633	0.3318	0.8266	0.0000
	Mannose	0.5584	0.1715	0.2204	0.0367
	Fucose	0.4191	0.2636	0.5908	0.0001
RP_0.5_	M_w_	1.5622	0.9491	0.0213	0.5383
	DM	1.2520	2.7252	0.3714	0.0043
	FA	1.2288	0.5233	0.3945	0.0030
	Phenols	1.0978	0.9115	0.5167	0.0003
	Protein	1.2457	0.6274	0.3777	0.0039
	Rhamnose	0.7618	0.9600	0.7672	0.0000
	Mannose	1.4590	0.4481	0.1464	0.0957
	Fucose	1.1170	0.7026	0.5297	0.0005

**Table 4:** The lack of effect of apple pectins on L929 cell proliferation and adhesion.

Sample	L929 Cells	
	Proliferation (% of Control)	Adhesion (% of Control)
Control	100.00 ± 3.83	100.00 ± 2.03
P_cel_	99.83 ± 4.64	100.61 ± 2.37
P_xyl_	102.19 ± 5.03	99.17 ± 3.83
P_cel + xyl_	100.41 ± 7.41	100.32 ± 2.94
P_acid_	97.91 ± 4.45	99.64 ± 2.79
P_commercial_	100.56 ± 6.55	102.10 ± 3.13

Data are expressed as mean ± SD of three independent experiments.

**Table:** A

Pectin	Pectin Features and Components					
	M_w_ (kDa)	GalA (%)	DM (%)	NS (%)	Protein (%)	Reactive with F–C Reagent (%)
P_cel_	589 ± 66	70.5 ± 1.8	66.3 ± 2.0	20.9 ± 1.1	3.11 ± 0.45	0.98 ± 0.14
P_xyl_	899 ± 79	61.1 ± 1.9	73.4 ± 2.4	29.8 ± 1.2	4.38 ± 0.38	1.34 ± 0.18
P_cel + xyl_	419 ± 34	74.7 ± 2.1	67.5 ± 2.3	17.9 ± 1.0	2.98 ± 0.42	1.01 ± 0.09
P_acid_	331 ± 42	59.9 ± 2.0	56.1 ± 2.5	31.1 ± 1.3	1.53 ± 0.26	0.71 ± 0.11
P_commercial_	378 ± 45	80.9 ± 2.4	56.9 ± 2.1	14.3 ± 0.7	0.78 ± 0.12	0.49 ± 0.08

**Table:** B

**Pectin**	**Neutral Sugars Content in Apple Pectin [g/100 g]**						
	Glucose	Galactose	Arabinose	Rhamnose	Xylose	Fucose	Mannose
P_cel_	6.69 ± 0.41	5.74 ± 0.13	4.57 ± 0.12	1.05 ± 0.04	2.27 ± 0.11	0.27 ± 0.02	0.40 ± 0.03
P_xyl_	8.06 ± 0.38	5.86 ± 0.21	8.99 ± 0.26	1.23 ± 0.03	2.49 ± 0.10	0.31 ± 0.02	2.91 ± 0.07
P_cel + xyl_	4.05 ± 0.29	5.27 ± 0.18	3.92 ± 0.19	1.38 ± 0.08	1.54 ± 0.06	0.25 ± 0.03	1.40 ± 0.04
P_acid_	17.50 ± 0.48	4.75 ± 0.23	5.29 ± 0.19	0.92 ± 0.03	2.18 ± 0.12	0.15 ± 0.02	0.30 ± 0.01
P_commercial_	2.35 ± 0.15	4.68 ± 0.19	5.20 ± 0.21	0.85 ± 0.02	1.15 ± 0.04	0.09 ± 0.01	0.00 ± 0.00

P_cel_—pectin extracted with 50 U endo-cellulase per 1 g dried apple pomace, P_xyl_—pectin extracted with 50 U endo-xylanase per 1 g dried apple pomace, P_cel + xyl_—pectin extracted with 50 U endo-cellulase and 50 U endo-xylanase per 1 g dried apple pomace, P_acid_—pectin extracted with H_2_SO_4_ at pH 2 and 85 °C; P_commercial_—commercial apple pectin (Pektowin S.A., Poland); M_w_—weight-average molecular weight, GalA—galacturonic acid, DM—degree of methylation, NS—total neutral sugars, F–C—Folin–Ciocalteu reagent.

## Data Availability

Data sharing not applicable.

## Sample Availability

Samples of the pectins are not available from the authors.
